# Cytokine and Microbiome Changes in Adolescents with Anorexia Nervosa at Admission, Discharge, and One-Year Follow-Up

**DOI:** 10.3390/nu16111596

**Published:** 2024-05-23

**Authors:** Larissa Käver, Clara Voelz, Hannah E. Specht, Anna C. Thelen, Lara Keller, Brigitte Dahmen, Nadia Andrea Andreani, Klaus Tenbrock, Ronald Biemann, Katrin Borucki, Astrid Dempfle, John F. Baines, Cordian Beyer, Beate Herpertz-Dahlmann, Stefanie Trinh, Jochen Seitz

**Affiliations:** 1 Institute of Neuroanatomy, RWTH Aachen University, Wendlingweg 2, 52074 Aachen, Germany; 2 West German Center for Child and Adolescent Health (WZKJ), University Hospital Cologne, Kerpener Str. 62, 50931 Cologne, Germany; 3 Department of Child and Adolescent Psychiatry, Psychosomatics and Psychotherapy, University Hospital RWTH Aachen, Neuenhofer Weg 21, 52074 Aachen, Germany; 4 Department of Child and Adolescent Psychiatry, Psychosomatics and Psychotherapy, LVR University Hospital Essen, Virchowstrasse 174, 45147 Essen, Germany; 5 Max Planck Institute for Evolutionary Biology, August-Thienemann-Str. 2, 24306 Plön, Germany; 6 Institute for Experimental Medicine, Kiel University, Christian-Albrechts-Platz 4, 24118 Kiel, Germany; 7 Department of Pediatrics, Medical Faculty, RWTH Aachen University, Pauwelsstraße 30, 52074 Aachen, Germany; 8 Department of Pediatrics, Pediatric Rheumatology, Inselspital University of Bern, Freiburgstrasse 15, 3010 Bern, Switzerland; 9 Institute of Laboratory Medicine, Clinical Chemistry and Molecular Diagnostics, University of Leipzig, Paul-List-Straße 13/15, 04103 Leipzig, Germany; 10 Institute for Clinical Chemistry and Pathobiochemistry, Otto-von-Guericke University Magdeburg, Leipziger Str. 44, 39120 Magdeburg, Germany; 11 Institute of Medical Informatics and Statistics, Kiel University, Brunswicker Str. 10, 24105 Kiel, Germany

**Keywords:** anorexia nervosa, cytokines, IL-15, IL-1β, IL-6 (TNF-α), gut microbiome

## Abstract

Anorexia nervosa (AN) is a severe eating disorder that predominantly affects females and typically manifests during adolescence. There is increasing evidence that serum cytokine levels are altered in individuals with AN. Previous research has largely focused on adult patients, assuming a low-grade pro-inflammatory state. The serum levels of the cytokine tumour necrosis factor-alpha (TNF-α), interleukin (IL)-1β, IL-6 and IL-15, which are pro-inflammatory, were examined in 63 female adolescents with AN and 41 age-matched healthy controls (HC). We included three time points (admission, discharge, and 1-year follow-up) and investigated the clinical data to assess whether the gut microbiota was associated with cytokine alterations. Relative to the HC group, serum levels of IL-1β and IL-6 were significantly lower during the acute phase (admission) of AN. IL-1β expression was normalised to control levels after weight recovery. TNF-α levels were not significantly different between the AN and HC groups. IL-15 levels were significantly elevated in patients with AN at all time points. We found associations between cytokines and bodyweight, illness duration, depressive symptoms, and the microbiome. In contrast to most findings for adults, we observed lower levels of the pro-inflammatory cytokines IL-1β and IL-6 in adolescent patients, whereas the level of IL-15 was consistently increased. Thus, the presence of inflammatory dysregulation suggests a varied rather than uniform pro-inflammatory state.

## 1. Introduction

Anorexia nervosa (AN) is a severe psychiatric disorder characterised by reduced food intake, leading to low bodyweight, disrupted body perception, hyperactivity, and anxiety regarding weight gain [1,2]

AN usually manifests during adolescence, a developmentally sensitive period, predominantly affecting girls and young women (male-to-female ratio from 1:10 to 1:20) [3]. It is associated with substantial morbidity and exhibits the highest mortality risk among mental disorders [1,4,5,6]. Nonetheless, its exact aetiology remains unclear, highlighting the urgent need to investigate its underlying pathophysiology, mental health comorbidities, and perpetuating factors to enable the development of new causally targeted therapeutic options.

The aetiology of AN is considered to be multifactorial, being affected by genetics [7], epigenetics [8], neurobiology, [9,10] cognition [11,12], and psychosocial and societal factors [13]. Emerging findings indicate a potential association between inflammation and several of the mental health disorders that frequently co-occur with AN, such as depression or anxiety [14,15]. In individuals with depression, the most common comorbidity in AN, circulating levels of pro-inflammatory cytokines such as interleukins (IL-1β, IL-6) and tumour necrosis factor-alpha (TNF-α) are significantly elevated, as shown by a meta-analysis of studies involving 5166 patients [16,17]. Cytokines, crucial intercellular signalling molecules in the immune system, are produced by various cells, including microglia and astrocytes in the brain, macrophages in the periphery, muscle cells, and adipocytes [18,19,20,21]. These molecules influence processes that are involved in the development of AN, such as the regulation of appetite, food intake and bodyweight, as well as the control of mood and cognition [22,23,24,25,26,27]. 

A low-grade pro-inflammatory state has recently been described in individuals with AN, although this has predominantly been studied in adult patients [28,29,30]. Serum concentrations of circulating pro-inflammatory cytokines (IL-1β, IL-6, TNF-α) are elevated in patients with AN relative to healthy controls (HCs), as shown by two meta-analyses [28,31] that included 22 studies (*n* = 924 patients) [28] and 23 studies (*n* = 577 patients) [31].

However, recent investigations of circulating cytokine levels in patients with AN have revealed inconsistent results for adults, throwing into question the assumption of a uniform pro-inflammatory status in patients with AN. Himmerich et al. [19] (*n* = 27 patients) and Caroleo et al. [32] (*n* = 90 patients) reported no significant differences in serum cytokine levels between adult patients and HCs. Conversely, a study by Keeler et al. [33], which included correction for confounding factors (age, ethnicity, smoking status, and the use of psychopharmacological medication), identified significantly lower levels of IL-6 in acute AN patients and TNF-α in patients that had recovered from AN. Similarly, contradictory results were reported for acutely ill adolescent patients with AN [34,35]. Notably, most of these studies were cross-sectional rather than longitudinal, and were thus unable to differentiate individual disease trajectories and distinguish between hunger-induced states and the effects of disease-related traits.

Recent studies have indicated that the gut microbiome, influenced by environmental stimuli such as altered dietary intake, plays a role in regulating bodyweight and modulating inflammatory processes [36,37,38]. The gut microbiota influences brain function and behaviour via the gut–brain axis [39]. These processes are implicated in AN and thus inspire further research into the microbiome, particularly regarding their relationship with the inflammatory status of acutely ill patients. A comprehensive study by Fan et al. [40] demonstrated that weight gain was significantly reduced in germ-free mice that received faecal transplants from patients with AN when they received limited food. The study also highlighted the potential role of microbiome dysbiosis in the pathophysiology of AN, although the relationship between this and inflammation was not investigated. 

Further research is required to better understand the involvement of pro-inflammatory cytokines in the pathophysiology of AN, and their association with the microbiome, in adolescent patients. Adolescents with AN often experience shorter illness durations than adults with AN and require fewer treatment interventions. This research could facilitate a more focused examination of the origins of AN and of the fundamental mechanisms involved.

A preliminary study from our group found that adolescent patients with AN exhibited significantly reduced levels of the pro-inflammatory markers IL-1β and IL-6, in contrast to the findings for adults. Further, we examined the links between these cytokines and the microbiome, finding that IL-1β levels were positively associated with *Bacteroides* abundance and IL-15 levels were positively associated with *Romboutsia* abundance, whereas negative associations were observed between IL-15 levels and *Anaerostipes* abundance and between TNF-α levels and uncultured (uncl.) *Lachnospiraceae* abundance [41].

This study addresses this gap by extending our previous study by adding 41 adolescent patients and 19 sex- and age-matched controls to our original cohort and adding a supplementary observation time point (1-year follow-up). Examining the individual course of the disease with an increased cohort size and including a longer-term perspective could help to answer the following research questions: I. Are inflammatory cytokines altered during acute AN in adolescents with AN, and if so in what way? II. Do these changes represent a reversible effect of starvation or an enduring disease-relevant trait? III. Can associations between changes in the microbiome and in cytokine levels be observed in order to explore their influences on the gut–brain axis? Answering these questions may help us to understand the pathophysiology of AN in adolescents and to develop appropriate individualised treatment options in the future. 

## 2. Materials and Methods

### 2.1. Participants and Study Design

Our study cohort consisted of 63 adolescent female patients diagnosed with typical or atypical AN who were treated as inpatients and recruited at the University Hospital RWTH Aachen (Germany) between September 2016 and January 2019. This study focused exclusively on females due to historical perceptions of AN as primarily affecting women. All of the participants were part of the Microbiome and Anorexia Nervosa (MaAN) Project. The time points considered were inpatient hospital admission, discharge to outpatient treatment, and 1-year follow-up (after admission). The following criteria were used for assessing inclusion: a diagnosis of AN according to DSM-5, female gender, and an age ranging from 12 to 18 years. Exclusion criteria included the use of antibiotics or probiotics after a time point of 4 weeks prior to enrolment, IQ < 85, inadequate proficiency in the German language, the presence of severe comorbid mental disorders, and significant gastrointestinal or metabolic conditions, such as coeliac disease or diabetes mellitus. The same exclusion criteria were applied to healthy volunteers, with the additional requirement of having a body weight above the 80th or below the 20th percentiles, which was adjusted for age in terms of body mass index (BMI-SDS); the absence of current psychiatric disorders; and a lack of a history of eating disorders. The multimodal inpatient treatment program was designed to address psychoeducation, body weight restoration, the improvement of eating behaviors, and the management of comorbid symptoms. It encompassed both individual and group psychotherapy sessions, close communication and collaboration with parents as co-therapists, supervised mealtimes, and comprehensive support from a multidisciplinary team comprising physicians, psychologists, nurses, nutritionists, and educators, as well as occupational, music, and physiotherapists, within a specialized 12-bed ward. Target weight goals were determined on an individual basis, taking into consideration pre-onset BMI percentiles (typically around the 20th percentile) as well as the onset of menses during the course of treatment.

Specht et al. published cytokine data from 22 of these patients at admission and discharge and from 19 HCs [41]. The patients were aged 12–20 years (mean 16.07 ± 1.81 years), with a mean body mass index (BMI) value of 15.95 ± 2.08 kg/m² at admission. Diagnoses were made according to the Diagnostic and Statistical Manual of Mental Disorders, version 5 (DSM-5) [2]. The mean duration of illness (time since diagnosis) was 18.38 ± 15.22 months, and the mean duration of inpatient treatment was 4.5 months. We collected clinical information for all patients and HCs, including age-adjusted standardised BMI (BMI-SDS), based on German population reference data from the KiGGS study [42]. Each participant completed The Eating Disorder Inventory 2 (EDI-2) [43] and Beck Depression Inventory-II (BDI-II) [44] self-report questionnaires. The patients underwent the Eating Disorder Examination (EDE, first German edition 2016) [45], a semi-structured eating disorder interview. This study started with 63 patients at admission, 50 of whom were available for analysis at discharge; cytokine data at 1-year follow-up were available for 45 patients. The sample size decreased by 13 between admission and discharge, mostly due to patients being discharged rather quickly before another blood draw could be initiated, and fell by 5 participants between discharge and the 1-year follow-up due to lack of motivation to participate in another visit. As healthy controls, we included 41 age-matched, normal-weight, healthy female adolescents with no self-reported history of mental illness. These controls were recruited for this study and were not hospitalized for any other concern. Written consent for participation was obtained from all participants and their legal guardians. Approval was granted by the local ethics committee of the RWTH Aachen University, and this study was conducted in accordance with the Declaration of Helsinki. Table 1 summarises the clinical parameters of patients and HCs.

### 2.2. Sample Collection

Fasting blood samples were collected from patients and HCs between 07:00 and 10:00 am at each time point. The samples were immediately centrifuged, and the serum was stored at −80 °C until use to limit proteolytic effects. After thawing at room temperature, inflammatory marker levels (for IL-15, IL-6, IL-1β, and TNF-α) were quantified in two batches using highly sensitive quantitative sandwich immunoassays (R&D Systems, Minneapolis, MN, USA) at the Institute of Clinical Chemistry and Pathobiochemistry, Otto von Guericke University Magdeburg (Magdeburg, Germany). The detection limit for IL-15 (cat. no. D1500) was a mean minimum detectable dose (MDD) of 2 pg/mL; for IL-6 (cat. no. HS600C), there was an MDD of 0.031 pg/mL; for IL-1β (cat. no. HSLB00D), there was an MDD of 0.033 pg/mL; and for human TNF-α (cat. no. HSTA00E), there was MDD of 0.022 pg/mL. This procedure based on the manufacturer’s specifications. 

Stool samples were collected upon admission to the hospital, at discharge, and at a 1-year follow-up. Disposable paper faeces catchers (The Feces Catcher, Tag Hemi VOF, Zeijen, The Netherlands) were used; a pea-sized sample was taken from two different parts of the faeces sample and transported to a sterile plastic container. Samples were stored at −80 °C until use. All stool samples from the patients and healthy controls were shipped on dry ice to collaborators at the University and Max Planck Institute in Kiel (Germany) for DNA extraction (using a DNeasy Power Soil Kit; Qiagen, Hilden, Germany) and sequencing. The resulting microbiome data, which were recently published by Andreani et al. [46], also served as the basis for this study. Microbial community characteristics were analysed by investigating the V1V2 region of the 16S rRNA gene. We focused on variation among the three time points in the abundance of the core genera (i.e., those present in ≥25% of individuals, with a relative frequency ≥1%). This method is described in more detail by Andreani et al. [46].

### 2.3. Statistical Analysis

Standard curves were used to determine the absolute levels (pg/mL) of each inflammatory marker. All subsequent statistical analyses were performed using SPSS Statistics 29.0 (IBM Corp., Armonk, NY, USA). To account for the skewed distributions of the cytokine values, the absolute values were logarithmically transformed, following the approach of Dalton et al. [47,48]. For values below the detection limit, we assumed that there was a value of 0.0001 before logarithmic transformation. Because the inflammatory parameters were quantified in two different batches, we first *z*-log-standardised the data. For this transformation, the mean value for each batch was subtracted from the log-transformed value for the sample and then divided by the standard deviation. Because of the unequal number of patients and controls, only the mean value and standard deviation were used for the HCs. To compare cytokine levels at the three time points between the patient and HC groups, the non-parametric Mann–Whitney U test was applied. All tests were conducted with the two-sided significance level set of α = 0.05.

To examine the intercorrelations between different cytokines and their associations with clinical variables (BMI-SDS, BDI-II, EDI-2, illness duration) over time, Spearman’s rank correlations were performed at each time point. Spearman’s rank correlations were also used to quantify the associations of delta ∆ (intra-individual changes between two time points) for the cytokines and for the same clinical variables as those mentioned above (delta ∆: follow-up minus admission/discharge or discharge minus admission).

Finally, we examined associations between cytokine levels and the abundance of gut microbiome genera that differed significantly between patients with AN and HC at admission. These genera were *Acinetobacter*, *Anaerostipes*, *Anaerotruncus*, *Bacteroides*, *Bilophila*, *Blautia*, *Collinsella*, *Desulfovibrio*, *Dialister*, *Eisenbergiella*, *Erysipelotrichaceae_UCG_003*, *Faecalibacterium*, *Family XIII AD3011 group*, *Family XIII UCG 001*, *Lachnospiraceae ND3007* group, *Legionella*, *Limnobacter*, *Ralstonia*, *Ruminococcacaea UCG 003*, and *Ruminococcacaea UCG 009* (Table 2). To effect size measure, rank-biserial correlation coefficient was calculated as *ρ* = 1 − (2U)/(n_1_ × n_2_). Only nominally significant associations were included (multiple comparison correction was not performed). To limit the number of comparisons, owing to the large interindividual differences in microbiome composition among the patients, we focused on the longitudinal correlations between the time of admission and discharge and between admission and 1-year follow-up. This allowed us to compare differences in cytokine levels and taxon abundance. Spearman’s correlation analysis was used to examine all monotonic relationships.

## 3. Results

### 3.1. Cytokine Levels

The clinical characteristics of the patients with AN are summarised in Table 1. As expected, patients with AN had significantly lower BMI values, worse eating disorder symptoms (based on EDI-2), and worse depression scores (based on BDI-II) upon admission than HCs. Although these scores, on average, decreased over time for each patient, they did not reach the levels seen in the HCs, either at discharge or at 1-year follow-up. 

**Figure 1:**
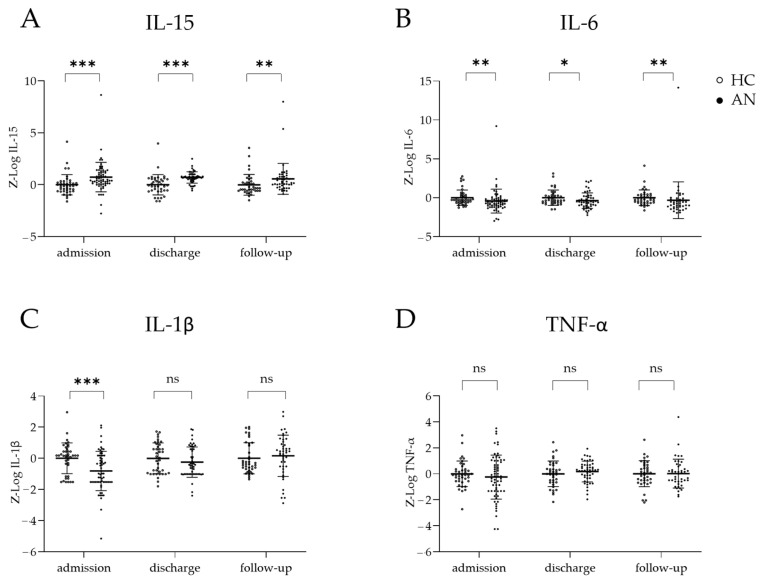
Concentrations–of cytokines in patients with anorexia nervosa (AN) and in healthy controls (HCs) at admission, discharge, and 1-year follow-up. Boxplots of serum cytokine levels, including mean and standard errors, comparing the AN and HC groups at admission, discharge, and 1-year follow-up for (**A**) IL-15, (**B**) IL-6, (**C**) Il-1β, and (**D**) TNF-α. Cytokine concentrations were log-transformed and batch-corrected via z-standardisation. Mann–Whitney U tests were performed to compare cytokine levels at each time point owing to their non-normal distributions. *ns* = not significant * *p* < 0.05, ** *p* < 0.01, *** *p* < 0.001.

At admission, relative to the HCs, the patients exhibited significantly higher serum IL-15 levels (*p* < 0.001) and lower serum IL-6 (*p* = 0.005) and IL-1β (*p* < 0.001) levels, whereas pairwise comparisons revealed no significant differences in TNF-α (*p* = 0.850) levels (Figure 1A–D). At discharge, serum IL-15 remained significantly elevated in the patients (*p* < 0.001), while IL-6 remained significantly reduced (*p* = 0.043). IL-1β and TNF-α were not significantly different relative to the HCs (*p* = 0.245 and *p* = 0.258, respectively) (Figure 1A–D). At the 1-year follow-up, the differences between the groups were significant for serum IL-15 (*p* = 0.003) and IL-6 (*p* = 0.002), but not for TNF-α (*p* = 0.639) and IL-1β (*p* = 0.214).

### 3.2. Correlations between Cytokines and Associations with Clinical Variables over Time

**Figure 2:**
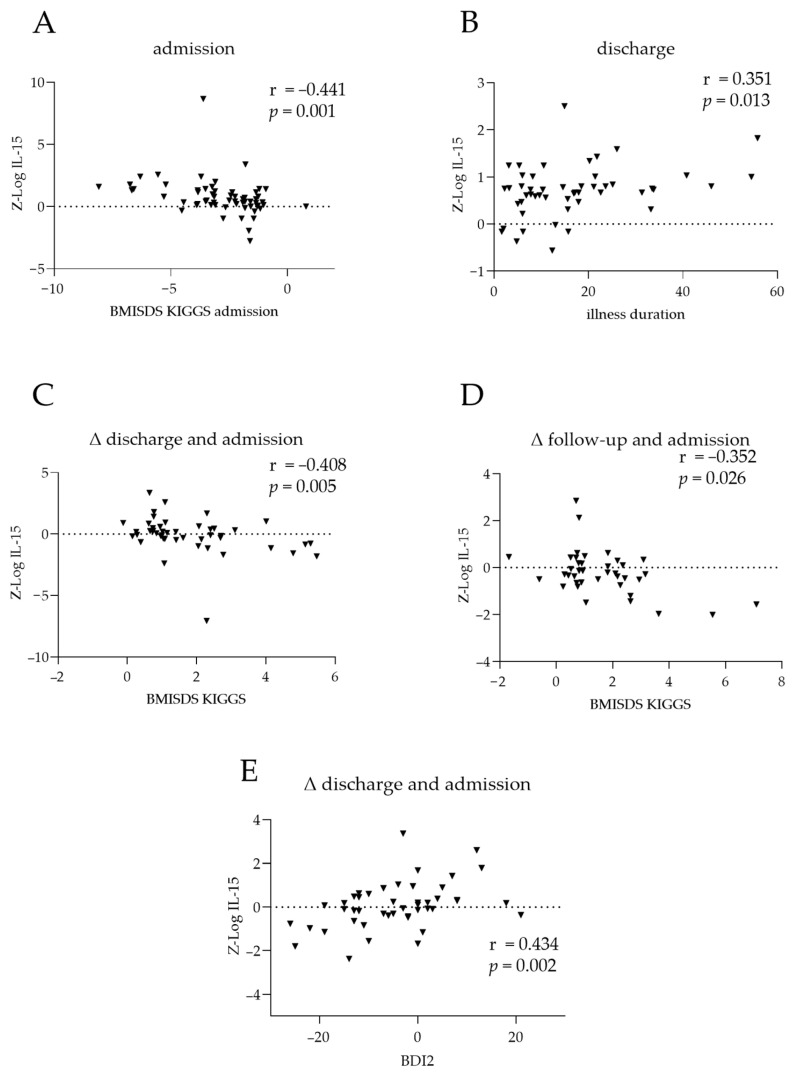
Clinical associations of IL-15 and TNF-α in patients with anorexia nervosa. Correlations between IL-15 at admission and (**A**) body mass index standard deviation scores (BMI-SDS) at admission and (**B**) illness duration. Differences in IL-15 between (**C**) discharge and admission or (**D**) 1-year follow-up and admission, compared to the corresponding changes in BMI-SDS. (**E**) Correlation between changes in IL-15 levels and changes in BDI2 scores between discharge and admission. The dotted line represents the position of zero. The triangles show individual values of the patients. Cytokine levels were log-transformed via z-standardisation and batch-corrected. r: Spearman’s correlation coefficient.

At admission, serum IL-15 and BMI-SDS were negatively correlated (r = −0.441; *p* = 0.001; Figure 2A). At discharge, serum IL-15 and duration of illness were positively correlated (r = 0.351; *p* = 0.013; Figure 2B). Within patients, changes in IL-15 and BMI-SDS from admission to discharge were negatively correlated (r = −0.408; *p* = 0.005; Figure 2C), as were changes from admission to the 1-year follow-up (r = −0.352; *p* = 0.026; Figure 2D). Changes in IL-15 and BDI-II score from admission to discharge were positively correlated (r = 0.434; *p* = 0.002; Figure 2E), indicating that a decline in IL-15 serum levels was associated with fewer depressive symptoms in patients. 

### 3.3. Associations with Intestinal Microbiota

**Figure 3:**
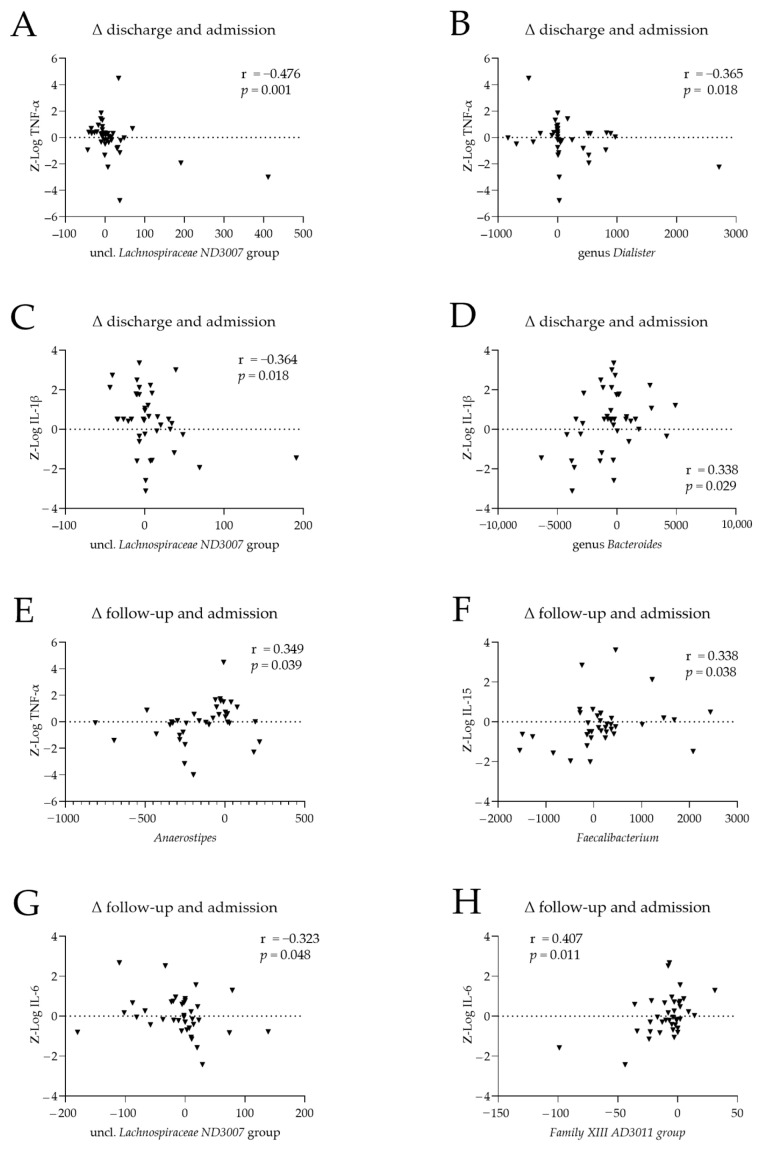
Associations between cytokine levels and the abundance of microbiome genera in patients with anorexia nervosa (AN). Differences between cytokine levels and microbiome genera abundances at (**A**–**D**) discharge and admission and (**E**–**H**) follow-up and admission, for TNF- α (**A**,**B**,**E**), IL-15 (**F**), IL-1β (**C**,**D**), and IL-6 (**G**,**H**). Cytokine levels were log-transformed and batch-corrected. r: Spearman’s correlation coefficient.

Within patients, we examined associations between changes in cytokine levels and changes in the abundance of the microbiome genera that differed significantly between patients with AN and HC at admission (Table 2). Changes from admission to discharge and admission to the 1-year follow-up were considered. Spearman’s correlation analysis revealed 13 nominally significant cytokine–microbiome associations, involving nine genera (Figure 3; Table A1). Changes from admission to discharge in cytokine levels were negatively associated with the corresponding changes in uncl. *Lachnospiraceae ND3007* abundance (r = −0.476, *p* = 0.001; Figure 3A) and *Dialister* abundance (r = −0.365, *p* = 0.018; Figure 3B). Changes in IL-1β levels between admission and discharge were negatively correlated with the corresponding changes in uncl. *Lachnospiraceae ND3007* abundance (r = −0.364, *p* = 0.018; Figure 3C) and positively correlated with those in *Bacteroides* abundance (r = 0.338, *p* = 0.0296; Figure 3D). Changes between admission and follow-up in TNF-α and IL-15 levels were positively correlated with the corresponding changes in *Anaerostipes* abundance (r = 0.349, *p* = 0.039; Figure 3E), and *Faecalibacterium* abundance (r = 0.338, *p* = 0.038, Figure 3F); similarly, changes in IL-6 levels were negatively correlated with the corresponding changes in uncl. *Lachnospiraceae ND3007* abundance (r = −0.323, *p* = 0.048; Figure 3G) and positively correlated with those in *Family XIII AD3011* abundance (r = 0.407, *p* = 0.011; Figure 3H).

## 4. Discussion

This study examined the inflammatory status of individual adolescent patients with AN at admission, discharge, and 1-year follow-up. The aim was to examine whether the prevailing assumption that adult women with AN have a low-grade pro-inflammatory state also applies to adolescent patients. Further, we aimed to determine whether changes in cytokine levels during the acute stage of AN are a transient response to AN or a disease-related trait effect of starvation. The patients exhibited significantly elevated IL-15 levels at all time points. In contrast to the prior findings of elevated pro-inflammatory IL-1β and IL-6 levels in adults, these markers were significantly reduced during the acute phase of the disease in the adolescent patients with AN, with no significant differences in the levels of TNF-α. This points to a more complicated inflammatory state, involving dysregulation in adolescent patients, than the uniform pro-inflammatory state previously posited. This difference may be due to the significantly shorter illness duration in adolescent patients, the different behaviour of the still-developing immune system, or the fact that, owing to their age, adolescents come into contact with fewer pathogenic stimuli than adults. The reduction in IL-1β levels observed here appeared to reverse with weight gain, reaching levels similar to those in the HCs upon weight recovery. For patients with AN, correlation analysis revealed that serum IL-15 was negatively associated with bodyweight and positively associated with clinical severity indicators, supporting the validity and clinical relevance of our findings. Within patients, changes over time in microbial abundance were significantly correlated with the corresponding changes in cytokine levels. These findings suggest that the gut microbiota may participate in modulating cytokine production, which could potentially influence inflammatory pathways and offer opportunities for therapeutic strategies. However, further investigation is required to understand the underlying pathophysiology of this immune alteration.

### 4.1. Cytokines

The research on cytokines in AN, which has mostly considered adult patients, has yielded inconsistent results [33,49]. For adolescent patients, cytokine levels were examined in the preliminary study by Specht et al. [41] and in a study performed by Bernardoni et al. [50]. Our preliminary study analysed a subset of the subjects from the present study (22 patients and 19 age-matched controls) and analysed only the admission and discharge time points. Our current analysis, based on a larger patient cohort (63 patients and 41 controls), corroborates most of the results for the first two time points, and extends the analyses up to the 1-year follow-up.

IL-1β and IL-6 levels were reduced in patients with AN at admission and followed different trajectories. IL-1β levels increased during treatment, and by discharge they were not significantly different from those in the HC group, whereas IL-6 levels remained low. This was consistent with our preliminary results [41], but was contrary to most previous findings. Bernardoni et al. [50] observed no cross-sectional differences in IL-6 or TNF-α levels between groups or no longitudinal changes for these cytokines in patients following weight restoration. Although the study of Bernardoni et al. [50] was not longitudinal, its results were consistent with our current findings. In the two previous meta-analyses, including 22 (*n* = 924 patients) [28] and recently 23 (*n* = 577 patients) [31] studies for AN, increased levels of IL-6 and IL-1β were found. The findings of the two meta-analyses are mostly consistent with recent findings for adults: Himmerich et al. [19] observed elevated levels of IL-6 in 27 acutely ill adult patients with AN and no differences in IL-1β levels, comparing both to 11 HCs. Roubalova et al. employed a longitudinal study design with 52 patients and 67 HCs, and observed that IL-6 levels were elevated both before and after hospitalisation in patients with AN. The most recent systematic review on cytokine alterations in AN [35] reported elevated IL-6 levels in patients with AN, but again found no differences in IL-1β relative to the HCs. This was based on various mixed-model analyses of patients aged 13–47 years. Few prior studies have examined changes in the cytokine levels of adolescent female patients, who often have a shorter duration of AN. Contrary to our findings, Ostrowska et al. [34] observed elevated serum IL-6 and IL-1β levels in 59 female adolescents with acute-phase AN relative to those observed in 23 HCs. Similarly, Caso et al. [51] reported elevated plasma IL-1β levels in acutely ill adolescent patients. The differences in these findings could be due to the different materials analysed and the different sensitivities of the ELISA kits used. Nonetheless, the reduced IL-6 level that we observed in acute-phase AN and after treatment was consistent with that observed in a well-controlled study by Keeler et al. using blood from 58 women with AN (>18 years old) [52], and with the findings of a study by Nova et al. [53] using mononuclear cells (peripheral blood cells with a round nucleus) from 40 adolescent female patients with AN.

IL-1β expression, a pro-inflammatory cytokine generated in blood monocytes following exposure to antigens such as lipopolysaccharides (LPS), is induced by cytokines such as IL-6 and TNF-α [54]. In the context of the gastrointestinal tract, IL-1β is linked to inflammation in the intestines and infections resulting from bacteria, viruses, and protozoa [55]. IL-6, which is classified as an acute-phase signalling molecule, controls and influences various immunological pathways in response to infection or tissue damage [56]. It is produced by various cells such as macrophages and T cells, and by non-immune cells such as fibroblasts and endothelial cells [57]. Research based on animal models has highlighted the significant involvement of IL-6 in intestinal inflammation. In a mouse model, exposure to foetal IL-6 resulted in reduced numbers of Paneth cells (found in crypts of the small intestine) and mucus-producing goblet cells, changes that increased susceptibility to intestinal injury [58]. 

As pro-inflammatory cytokines are not generated exclusively by immune cells but also produced by adipocytes [59], one potential explanation for the diminished levels of IL-6 and IL-1β in patients with AN could be the reduction in this tissue induced by starvation. Notably, a positive correlation between BMI and IL-6 levels was previously observed in obese patients [60]. A significant reduction in IL-6 mRNA expression in the adipose tissue of adult patients with AN was reported [61]. However, considering that IL-6 and IL-1β levels were nor significantly correlated with BMI-SDS at any time point in our study, it is unlikely that bodyweight reductions were the main drivers of the reduced IL-6 and IL-1β that we observed. Other factors that could explain the reduced cytokine levels include changes in regulatory pathways owing to undernutrition, which may cause the body to reduce cytokine production to preserve energy. However, our findings of increased IL-15 and unchanged TNF-α levels contradict this energy-saving hypothesis.

Because patients with AN often experience amenorrhoea, a difference in IL-1β levels relative to the HC group can also occur owing to an increase in these levels in the HC group post ovulation [62]. Dietary habits (e.g., low-fat intake [63] or a vegan or ketogenic diet [64]) may affect cytokine levels in an anti-inflammatory [65,66] or pro-inflammatory manner [67]. The duration of illness could exert an additional impact: the adolescent patients with AN in our study exhibited significantly shorter illness durations (mean ± SD, 18.38 ± 15.22 months) than the adults (11.68 ± 12.2 months) reported in the work of Dalton et al. [48]. Age itself has been identified as being associated with elevated cytokine levels [68,69], potentially contributing to the lower cytokine levels observed in our investigation of adolescents. 

The increased levels of IL-15 seen over time are consistent with previous findings for adults and adolescents, suggesting a specific role for IL-15 in the pathophysiology of AN. IL-15, a member of the immunoregulatory cytokine family, appears to play a versatile role in the innate immune response. It serves multiple functions, including regulation of T cell responses, monitoring of tissue repair, and modulation of inflammatory responses [70,71,72]. IL-15 exhibits pro- or anti-inflammatory properties in various tissues. For example, it exerts beneficial anticancer effects by promoting natural killer cell activity and supporting CD8^+^ T cell survival [73], whereas it has detrimental effects in inflammatory bowel disease, inducing the production of other pro-inflammatory cytokines by activating macrophages [70,74,75].

Elevated levels of IL-15, a T cell growth factor, appear to influence the depressive state by modulating serotonergic transmission [76]. This is consistent with our finding of a positive association between IL-15 levels and BDI-II scores. This is further consistent with evidence of altered serotonin levels in patients with AN, in combination with depressive symptoms and sleep disturbances [77,78]. IL-15 is also known to have metabolic effects, stimulating anabolic processes by reducing fat content and increasing muscle mass [79]. The fact that IL-15 is released from muscles following physical activity, as is often observed in patients with AN, may explain its elevated levels observed here, although this creates a conflicting situation. While IL-15 is responsible for breaking down adipocytes, it also helps to maintain muscle mass in cases of acute illness. The elevated IL-15 levels found in adolescent AN may reflect an already described adaptive response to chronic starvation in order to prevent the loss of muscle mass [47,80]. This hypothesis is supported by the finding that patients with <20% body fat presented significantly higher IL-15 levels than those in the control group (with body fat mass ≥ 30%) [81]. This relationship was also reflected in our results, as we found associations with BMI-SDS over the course of this study, and no significant differences in IL-15 levels between the groups after correcting for bodyweight. 

Overall, our findings for adolescent patients with AN suggest that they do not exhibit a uniform low-grade pro-inflammatory state, as has been assumed to exist in adults with AN. Instead, there appears to be a more complex state of inflammatory dysregulation, with both increased and reduced levels of pro-inflammatory cytokines. This imbalance can potentially be influenced by anti-inflammatory drugs, thereby offering new targets for individualised therapeutic interventions in AN. Currently, inhibitors of pro-inflammatory cytokines, with anti-inflammatory effects, are available. Meta-analyses have shown that treatment with these inhibitors can lead to increases in bodyweight [82,83]. 

### 4.2. Gut Microbiota

There is ample evidence to suggest that the gut microbiome plays a crucial role in modulating the immune system [84]. The apparent state of gut dysbiosis in patients with AN [30,40,46,85] might contribute to the observed inflammatory dysregulation. Thus, we explored alterations between time points in cytokine levels and their associations with corresponding changes in bacterial taxon abundance for the core genera (those with significantly different abundances between the AN and HC groups at admission). This revealed 13 significant cytokine–microbiome associations (6 negative and 7 positive); of these associations, 12 were for the differences between admission and discharge, while only 1 was for the difference between admission and follow-up. The connection between psychiatric disorders and the gastrointestinal tract is regulated by the gut–brain axis [38,86], which favours bidirectional communication between the central nervous system (brain and spinal cord) and the enteric nervous system, including gut bacteria and their metabolites in the gastrointestinal tract [87]. Bacterial metabolites, such as short-chain fatty acids, which can cross the blood–brain barrier and influence enteric and brain cells, providing a direct link between these systems [88]. Inflammation and immune processes originating in the gut impact the brain via cytokines and migrating immune cells [89]. Recent studies have focused on elucidating the precise effects of the gut microbiota on distinct enteric target structures [39,90]. Andreani et al. [46] examined longitudinal microbiome changes in adolescent patients with AN, finding that bacterial taxon abundances at admission were prognostic of hospital readmission. For instance, higher *Sutterella* abundances were associated with higher body weight in patients at the 1-year follow-up. 

Here, abundances of taxa such as Dialister and Bacteroides were positively correlated with pro-inflammatory cytokine levels. This relationship was reported for Dialister in inflammatory conditions such as spondyloarthritis [91,92] and for Bacteroides in inflammatory bowel disease [93,94]. Here, the levels of most of the pro-inflammatory cytokines were negatively correlated with the abundance of microbial taxa known for their anti-inflammatory or beneficial properties, including uncl. Lachnospiraceae [95,96], Anaerostipes [97,98], and Faecalibacterium [99], providing supporting evidence of this potential interaction. Lachnospiraceae, recognised for its ability to produce butyrate, has been linked to beneficial effects on cardiovascular risk factors [100], and exerts anti-inflammatory effects against inflammatory bowel disease [101]. For patients with AN, Schulz et al. [102] identified elevated uncl. Lachnospiraceae abundance upon admission as being indicative of a shorter treatment duration, representing a positive outcome. Faecalibacterium exerts protective effects in AN and gastrointestinal disorders (including inflammatory bowel disease) by exhibiting reduced levels in the acute state of AN [103,104]. Thus, there seems to be a reciprocal relationship between the gut microbiota and inflammation in AN.

### 4.3. Strengths and Limitations

This study has several strengths. Its sample size is among the largest available to date, and it is the first to include a longitudinal follow-up at 1 year. This provides an opportunity to examine microbiome associations within subjects. 

Regarding its limitations, the cytokine data were acquired in different batches owing to the limited number of samples per ELISA plate and the two time points of the analysis, potentially introducing measurement bias. To minimise batch effects, we corrected for this during statistical analysis. The statistical analyses did not include additional lifestyle covariates. Although our study had a relatively large sample size, future research could benefit from larger cohorts, preferably from multiple sites, to help rule out regional microbiome differences and the influence of local food preferences. Larger sample sizes would facilitate adjustment for comorbidities and the potential influence of medications on cytokine alterations.

## 5. Conclusions

This present study is the first to analyse cytokine changes in adolescent patients with AN at three different time points, including after a 1-year follow-up period. IL-1β and IL-6 exhibited significantly lower levels during the acute phase at admission. Surprisingly, IL-1β, but not IL-6, reached levels similar to those in the HC group following weight recovery. Low bodyweight was negatively correlated with increased IL-15 serum levels at admission, whereas IL-15 was positively correlated with illness duration and depression scores. Changes in cytokine levels were negatively associated with the abundance of microbial taxa known for their anti-inflammatory or beneficial properties, consistent with the potential immunomodulatory roles of these microbes. Our study therefore contributes to a better understanding of the fundamental inflammatory regulation in adolescent patients with AN, who exhibited complex dysregulation of the inflammatory state, rather than simply a low-grade pro-inflammatory state, as previously assumed. Furthermore, the interplay between cytokine levels and the microbiome offers the potential to derive new mechanistic insights and develop therapeutic approaches for treating AN.

## Figures and Tables

**Table 1:** Demographic and clinical parameters of participants.

Clinical Data	AN			HC		
	Admission	Discharge	1-Year Follow-Up	Baseline	6 Month Follow-Up	1-Year Follow-Up
	*n* = 63	*n* = 50	*n* = 45	*n* = 41	*n* = 39	*n* = 39
Age (years)	16.07 (1.81)	16.31 (1.75)	16.89 (1.81)	16.32 (1.08)	16.68 (1.09)	17.35 (1.12)
	[12.00; 20.34]	[12.23; 19.91]	[13.10; 19.84]	[14.11; 18.46]	[14.44; 18.8]	[15.05; 19.42]
BMI (kg/m^2^)	15.95 (2.08)	18.92 (0.93)	19.04 (1.44)	20.67 (2.26)	20.90 (2.16)	21.20 (2.34)
	[12.5; 24.65]	[16.84; 20.96]	[15.85; 21.93]	[16.84; 26.07]	[16.89; 25.64]	[17.25; 25.94]
BMI-SDS (z-score)	−2.91 (1.74)	−0.95 (0.45)	−1.02 (0.7)	−0.36 (0.77)	−0.32 (0.73)	−0.29 (0.79)
	[−8.08; 0.70]	[−2.44; −0.2]	[−2.95; 0.25]	[−1.82; 1.15]	[−1.61; 1.05]	[−1.75; 1.2]
Pre-morbid weight BMI-SDS (z-score)	−0.38 (1.09)					
	[−4.24; 1.77]					
Illness duration (month)	18.38 (15.22)					
	[1.23; 71.97]					
EDI-2 (total score)	296.77 (58.46)	285.8 (62.92)	272.76 (55.15)	180.88 (29.78)	171.24 (25.55)	184.87 (32.57)
	[158; 428]	[127; 415]	[156; 392]	[123; 236]	[127; 226]	[133; 253]
BDI-II (total score)	24.18 (11.63)	19.27 (13.34)	17.24 (13.33)	4.7 (4.05)	3.24 (3.18)	4.63 (3.45)
	[0; 47]	[0; 54]	[0; 53]	[0; 17]	[0; 12]	[0; 11]
Medication SSRI/neuroleptics	*n* = 3	*n* = 25	*n* = 18	-	-	*n* = 4
	*n* = 9	*n* = 11	*n* = 8	-	-	-

Values are presented as mean (standard deviation) [minimum; maximum]. AN: anorexia nervosa; HC: healthy control; BMI: body mass index; BMI-SDS: body mass index standard deviation scores based on KiGGS data; BDI-II: Beck Depression Inventory; EDI-2: Eating Disorder Inventory; SSRI = selective serotonin reuptake inhibitor.

**Table 2:** Genus-level microbial abundance at admission.

Core Taxa	HC	AN	Rank-Biserial Correlation	AN vs. HC (Admission)
	Admission	Admission	Coefficient *ρ*	*p*-Value
*Acinetobacter*	0.00 (0.25) [0.00; 3.00]	0.00 (0.00) [0.00; 0.00]	−0.21	<0.001
*Anaerostipes*	24.50 (70.50) [10.00; 310.00]	150.00 (295.00) [0.00; 1595.00]	0.61	<0.001
*Anaerotruncus*	0.00 (0.00) [0.00; 1.00]	0.00 (2.25) [0.00; 237.00]	0.40	0.008
*Bacteroides*	2407.50 (2003.75) [306.00; 5957.00]	3173 (3599.25) [419.00; 6788.00]	0.37	0.036
*Bilophila*	0.00 (2.25) [0.00; 30.00]	6.00 (8.25) [0.00; 25.00]	0.48	0.005
*Blautia*	52.50 (137.50) [8.00; 492.00]	136.50 (209.50) [9.00; 695.00]	0.37	0.034
*Collinsella*	0.50 (6.00) [0.00; 41.00]	5.50 (19.75) [0.00; 295.00]	0.35	0.039
*Desulfovibrio*	0.00 (0.00) [0.00; 0.00]	0.00 (6.50) [0.00; 590.00]	0.30	0.022
*Dialister*	471.50 (680.25) [0.00; 1871.00]	25.00 (157.25) [0.00; 2023.00]	−0.58	<0.001
*Eisenbergiella*	0.00 (0.00) [0.00; 0.00]	0.00 (1.25) [0.00; 44.00]	0.30	0.022
*Erysipelotrichaceae_UCG_003*	21.00 (69.75) [0.00; 159.00]	129.00 (195.00) [0.00; 1195.00]	0.54	0.002
*Faecalibacterium*	610.00 (620.50) [88.00; 1207.00]	285.00 (317.75) [13.00; 2207.00]	−0.35	0.044
*Family XIII AD3011 group*	4.00 (6.25) [0.00; 11.00]	8.00 (13.75) [0.00; 127.00]	0.49	0.005
*Family XIII UCG 001*	4.00 (9.25) [0.00; 11.00]	0.00 (3.00) [0.00; 15.00]	−0.34	0.037
*Lachnospiraceae ND3007 group*	1.00 (9.50) [0.00; 27.00]	12.50 (39.00) [0.00; 355.00]	0.43	0.013
*Legionella*	0.00 (0.00) [0.00; 4.00]	0.00 (0.00) [0.00; 0.00]	−0.14	0.005
*Limnobacter*	0.00 (1.25) [0.00; 6.00]	0.00 (0.00) [0.00; 0.00]	−0.29	<0.001
*Ralstonia*	2.00 (4.50) [0.00; 18.00]	0.00 (1.00) [0.00; 65.00]	−0.29	0.048
*Ruminococcacaea UCG 003*	29.00 (52.75) [2.00; 81.00]	3.00 (15.00) [0.00; 115.00]	−0.66	<0.001
*Ruminococcacaea UCG 009*	0.00 (1.00) [0.00; 2.00]	0.00 (0.00) [0.00; 24.00]	−0.21	0.020

Values are reported as follows: the median and interquartile ranges are in round brackets, and minimum and maximum values are shown in square brackets (of the 20 most significantly altered taxa at admission; *p* < 0.05). Mann–Whitney U tests were used to test for differences between the groups. To measure effect sizes, the rank-biserial correlation coefficient r was calculated. Positive values of r indicate higher abundance in AN than in HC, while negative values of r indicate higher abundances in HC. AN: anorexia nervosa; HC: healthy control.

## Data Availability

The raw data can be obtained by contacting the data availability committee at kjp-data-access@ukaachen.de with a reasonable request.

## Appendix A

**Table A1:** Nominally significant delta-correlations for alterations of cytokines and microbiota at admission in patients with AN.

		IL-15		IL-6		IL-1β		TNFα	
		Δ Discharge and Admission	Δ Follow-Up and Admission	Δ Discharge and Admission	Δ Follow-Up and Admission	Δ Discharge and Admission	Δ Follow-Up and Admission	Δ Discharge and Admission	Δ Follow-Up and Admission
Anaerostipes	Correlation coefficient								0.349
	sig. (*p*-value)								0.034
	*n*								37
Anerotruncus	Correlation coefficient								0.562
	sig. (*p*-value)								<0.001
	*n*								37
Bacteroides	Correlation coefficient					0.338			
	sig. (*p*-value)					0.029			
	*n*					42			
Collinsella	Correlation coefficient				0.348				
	sig. (*p*-value)				0.032				
	*n*				38				
Dialister	Correlation coefficient							−0.365	
	sig. (*p*-value)							0.018	
	*n*							42	
Erysipelatoclostridium	Correlation coefficient			−0.345					
	sig. (*p*-value)			0.025					
	*n*								
Faecalibacterium	Correlation coefficient		0.338						
	sig. (*p*-value)		0.038						
	*n*		38						
Family XIII AD3011	Correlation coefficient	−0.320			0.407				0.427
	sig. (*p*-value)	0.039			0.011				0.008
	*n*	42			38				37
Lachnospiraceae ND3007	Correlation coefficient				−0.323	−0.364		−0.476	
	sig. (*p*-value)				0.048	0.018		0.001	
	*n*				38	42		42	

Cytokine levels were log-transformed and batch-corrected. Spearman’s correlation coefficient is reported.
